# Line-narrowing by polychromatic selective spin-locking in NMR

**DOI:** 10.5194/mr-7-135-2026

**Published:** 2026-10-02

**Authors:** Coline Wiame, Hadi Loutfi, Kirill Sheberstov, Geoffrey Bodenhausen

**Affiliations:** 1 Chimie Physique et Chimie du Vivant (CPCV, UMR 8228), Department of Chemistry, École Normale Supérieure, PSL University, Sorbonne University, CNRS, Paris, 75005, France

## Abstract

Selective Spin-Locking (SSL) of the magnetisation vectors of selected singlets or multiplets in high-resolution Nuclear Magnetic Resonance (NMR) spectra by mono- or polychromatic selective radio-frequency (RF) irradiation can reduce the line-widths to the limit given by homogeneous $T_{1\rho }$ relaxation. The resulting line-narrowing leads to an increase in signal height of spectra of ^1^H, ^19^F, ^31^P or other nuclei that is in principle only limited by the ratio $T_{1\rho }$ / $T_{2}$ *. At the same time, the scalar interactions that give rise to multiplets are decoupled, thus leading to an additional gain in signal height. The amplification factors allow one to use samples at low concentrations. Common one-dimensional (1D) experiments such as inversion recovery (IR) as well as the excitation and reconversion of Long-Lived States (LLS) by Spin-Lock Induced Crossing (SLIC) can significantly benefit from SSL.

## 1 Introduction

Line-widths in NMR spectra are often broad due to inhomogeneous broadening arising from susceptibility effects, sample heterogeneity, or imperfect static field homogeneity. The SHARPER (Sensitive, Homogeneous, And Resolved PEaks in Real time) method enables exceptionally narrow NMR resonances by suppressing dephasing arising from chemical shift evolution and magnetic field inhomogeneities during acquisition (Jones et al., 2017; Davy et al., 2022; Dickson et al., 2022). In its conventional form, however, SHARPER can refocus only one or two resonance frequencies, precluding simultaneous linewidth reduction across multiple chemically distinct spins. To achieve line-narrowing on multiple peaks, we have developed a method that we shall refer to as Selective Spin-Locking (SSL), which is based on our earlier work on “polychromatic decoupling” (Carnevale et al., 2012), where a number of short pulses with small flip angles are inserted in the intervals between the sampling points of the free induction decay (FID). Such short pulses are reminiscent of selective irradiation by Delays Alternating with Nutations for Tailored Excitation (DANTE) (Bodenhausen et al., 1976; Morris and Freeman, 1978), and Irradiation of Narrow Frequency Envelopes by Repeated Nutation and Orbiting (INFERNO) (Morris and Freeman, 1978), which are selective excitation methods that are widely used in NMR spectroscopy which use a comb of equidistant low-power RF pulses to achieve selective excitation of specific nuclear resonances. A suitable choice of the phases of these short pulses allows one to generate an irradiation spectrum where multiple peaks of interest are hit simultaneously. The combs of short pulses have the effect of *spin-locking* the magnetisation vectors belonging to the irradiated singlets or multiplets in their *local rotating frames*, thus preventing the phase dispersion due to the static field inhomogeneity and to *J-couplings*. In this case, the decay of the spin-locked transverse magnetisation will be determined by $\mathrm{exp}\{-t/T_{1\rho }\}$ rather than by $\mathrm{exp}\{-t/T_{2}^{*}\}$ so that the narrowest possible line-width, $\Delta \nu_{\min}^{\mathrm{SSL}}$ , is determined by the dissipation of the selectively spin-locked magnetisation in the rotating frame (Redfield, 1955), i.e., $\Delta \nu_{\min}^{\mathrm{SSL}}=1/\pi T_{1\rho }$ .

Many common one-dimensional (1D) experiments designed to probe molecular motions can benefit from Selective Spin-Locking (SSL). Inversion Recovery (IR) experiments provide longitudinal relaxation times ( $T_{1}$ ), enabling one to estimate rotational correlation times ( $\tau_{c}$ ), among other parameters. Carr-Purcell-Meiboom-Gill (CPMG) spin-echo experiments yield transverse relaxation times ( $T_{2}$ ), allowing for the assessment of chemical exchange effects and other line-broadening mechanisms, such as scalar relaxation of the second kind. Other measurements that use pulsed field gradients (PFG) in order to determine diffusion coefficients can also benefit from SSL. Beyond 1D experiments, SSL can be combined with the many two-dimensional (2D) experiments used in NMR. For many studies, multiplet structures are essentially a nuisance, making the use of SSL valuable.

## 2 Mono- or Polychromatic Selective Spin-Locking (SSL)

The pulse sequence in Fig. 1 shows how mono- or polychromatic SSL during the acquisition of a Free Induction Decay (FID) is applied after a preparation period that can represent any arbitrary NMR experiment.

**Figure 1:**
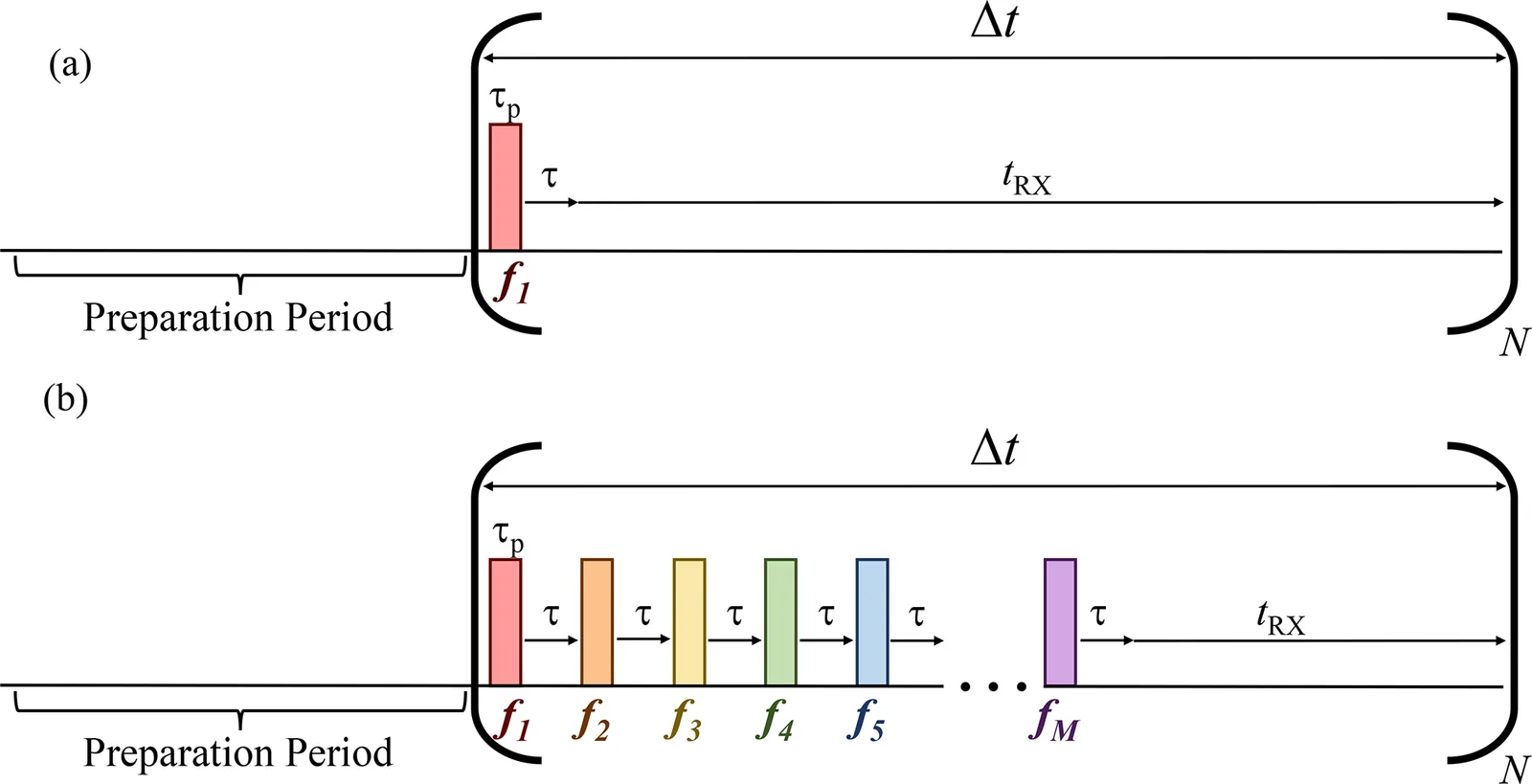
Schemes for **(a)** mono- or **(b)** polychromatic SSL, showing how a series of M short pulses separated by delays τ and associated with chemical shifts $f_{m}$ (where m=1,2,…,M ), each having a duration $\tau_{p}$ , can be applied during each interval Δt (typically equal to 152 µs ), to generate an FID. During the interval “ $t_{\mathrm{RX}}$ ” the receiver is activated to record sampling points, usually with oversampling. Each sequence of M short pulses is repeated N times, until the total number of points has been measured. Typically, N=32 k =32 768 in our experiments, so that the length of the FID is NΔt=4.92 s. The preparation period may consist of a simple π/2 excitation pulse, a $T_{1}$ inversion recovery sequence, a $T_{2}$ CPMG echo sequence, or indeed one of the very large number of two-dimensional (2D) experiments where the SSL sequence can be used in the detection interval. For in vivo or in vitro Magnetic Resonance Spectroscopy (MRS), the sequence must be preceded by a method to select a volume of interest.

In SSL experiments, the preparation period usually includes a “hard” non-selective π/2 pulse, typically of 10 µs for a peak RF amplitude $\omega_{1}/(2\pi )=25$ kHz, followed by the SSL sequence comprising M short equidistant pulses applied during the acquisition block. The “soft” SSL pulses, separated by intervals τ=1.1 µs have a duration $\tau_{p}$ , which ranges typically from 1.5 to 7 µs , with a peak RF amplitude on the order of 2 kHz. As a result, the individual pulses have small nutation angles in the range 1°<β<10° . Each sequence of M short pulses $\tau_{p}^{m,n}$ with m=1,2…M and n=1,2…N constitutes a “comb” in the manner of DANTE (Carnevale et al., 2012; Bodenhausen et al., 1976; Morris and Freeman, 1978). The phase of the π/2 pulse must be alternated between x and -x , to remove artefacts arising from RF breakthrough. The phases of SSL pulses are initially applied along y , so that they are shifted by 90° with respect to the π/2 pulse. The phase of the receiver must be alternated. These considerations entail that SSL experiments require a minimum of two scans.

To irradiate M chemical shifts $f_{m}$ simultaneously, one must apply a superposition of pulses m=1,2,…,M , incrementing their phases from one interval Δt to the next ( n=1,2,…N ) by phase-shifts $\Delta \Phi_{m,n}=n\Delta \varphi_{m}$ , where the “phase roll” $\Delta \varphi_{m}$ can be calculated by the following equation:

1 $$\Delta \varphi_{m}=2\pi \Delta t\Delta f_{m}$$

where, as shown in Fig. 2, $\Delta f_{m}$ corresponds to the difference in frequency (in Hz) between the RF carrier frequency and the m th peak of interest. Using $\Delta f_{m}$ , the phase shift required to apply the m th short pulse, $\Delta \varphi_{m}$ , to the m th peak of interest can be calculated using Eq. (1). For monochromatic SSL (Fig. 1a), the carrier frequency can be set to be on-resonance with the peak of interest so that $\Delta \varphi_{m}=0$ °.

**Figure 2:**
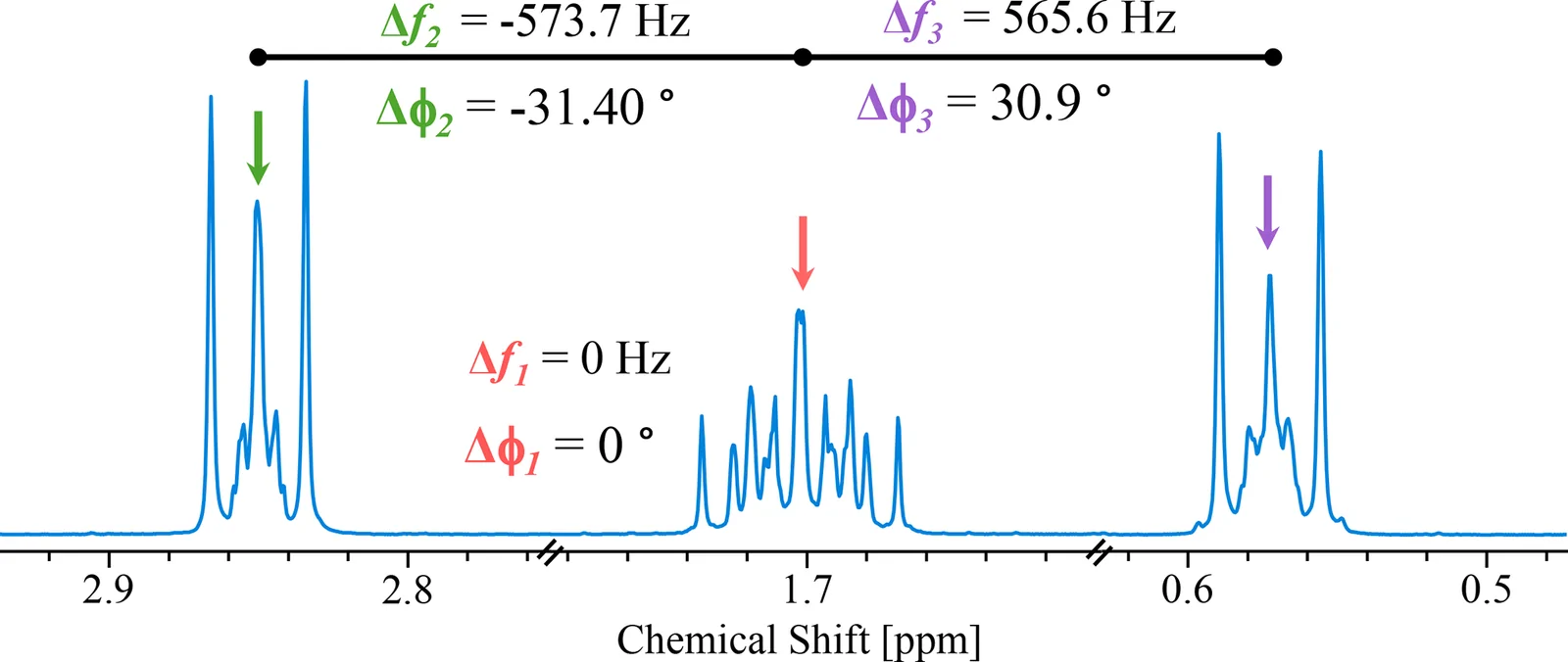
One-dimensional (1D)^1^H spectrum showing peaks corresponding to the three CH_2_ groups of 200 mM sodium trimethylsilylpropanesulfonate (DSS) in 100 mM phosphate buffer solution (PBS, pH 7.2) in D_2_O at 11.7 T (500 MHz for ^1^H) showing the phase incrementation required for trichromatic SSL ( M=3 ) with Δt=152 µs , the carrier frequency being set on the central peak (red arrow). The green and purple arrows designate the positions irradiated using phase increments calculated with Eq. (1) to coincide with the central lines of the high- and low-frequency multiplets.

Signal acquisition occurs during a short time interval, designated by the delay “ $t_{\mathrm{RX}}$ ” in the sequences, during which the receiver is activated in so-called oversampling mode. As illustrated in Fig. 1, the delay Δt remains the same for mono- or polychromatic sequences; the variable delay is “ $t_{\mathrm{RX}}$ ” which becomes shorter for experiments with a larger number M of SSL pulses. The total number of points is observed after N repetitions of the SSL sequence (Fig. 1, bracketed section). Typically, N can be as large as 32 k or more. One can replace the intervals marked “ $t_{\mathrm{RX}}$ ” in Fig. 1 by empty delays, in which case the line-narrowing method can be inserted in one or several evolution intervals of n -dimensional experiments.

The peak RF field amplitude of the short pulses is typically $\omega_{1}/(2\pi )=2$ kHz. The average RF field strength is proportional to the duty cycle:

2 $$\langle \omega_{1}/(2\pi )\rangle =\omega_{1}/(2\pi )\tau_{p}/\Delta t$$

In our work, the duty cycle is typically $\tau_{p}/\Delta t=0.0125$ so that $\langle \omega_{1}/(2\pi )\rangle$ =25 Hz in our examples, corresponding to ca. 2 mW for each of the M interleaved DANTE sequences, well below the maximum power that can safely be applied to commercial solution-state NMR probes. The average RF field amplitude should be comparable to $1/(\pi T_{2}^{*})$ if one wishes to narrow a single inhomogeneously broadened line, and comparable to J if one wishes to collapse a multiplet at the same time.

## 3 Monochromatic SSL for Line-Narrowing in the Absence of Couplings

The long-range J -couplings of the nine magnetically equivalent methyl protons in sodium trimethylsilylpropanesulfonate (DSS) to neighbouring protons are negligible, so that one can focus on the ability of SSL to overcome the effects of $B_{0}$ inhomogeneity. A conventional proton spectrum was measured after a π/2 pulse, using a 5 mm Bruker NEO iProbe at 11.7 T (500 MHz for ^1^H), where a full width at half-height (FWHH) of approximately 1 Hz was achieved by rough shimming (Fig. 3, blue line). By deliberately mis-setting the $z_{1}$ -gradient of Bruker's room temperature shim system, the line was broadened to ca. 40 Hz (Fig. 3, light blue line).

As mentioned before, the smallest line-width that can be achieved is determined by the transverse relaxation time in the rotating frame, $T_{1\rho }$ (Redfield, 1955). For the methyl protons of DSS, $T_{1\rho }=3.04$ s at 298 K and 11.7 T, as measured by applying the INFERNO method (Morris and Freeman, 1978) with increasing durations between 1 ms and 40 s after an initial π/2 excitation pulse. Therefore, the theoretical minimum line-width that can be achieved in the case of these methyl protons is $\Delta \nu_{\min}=1/\pi T_{1\rho }=0.105$ Hz. The $T_{1}$ of these protons is equal to 3.13 s; the fact that $T_{1\rho }$ and $T_{1}$ are nearly equal shows that the extreme narrowing regime ( $\omega_{0}\tau_{c}<1$ ) is fulfilled to a good approximation.

The monochromatic SSL sequence (Fig. 1a) was applied to the middle of the inhomogeneously broadened resonance of the methyl groups of DSS, setting the duration of the short pulses $\tau_{p}=2.85$ µs (nutation frequency =2.0 kHz for ^1^H), applied within Δt=152 µs (spectral width 6579 Hz), to achieve an average RF amplitude $\langle \omega_{1}/(2\pi )\rangle =37.5$ Hz. The monochromatic SSL leads to a narrow peak (dark blue line in Fig. 3) with a Δν=0.23 Hz, about 2.2 times broader than the theoretical limit $\Delta \nu_{\min}$ = 0.105 Hz. This demonstrates that SSL with very weak RF fields can overcome severe inhomogeneous broadening. Monochromatic SSL was also applied to the well-shimmed proton signal (Fig. 3, blue line) with Δν=1.0 Hz. The resulting line-narrowed spectrum, not shown in Fig. 3, also led to Δν=0.23 Hz. The line-narrowed peaks resulting from both the inhomogeneously broadened peak and from the well-shimmed peak show the same line-widths illustrating the ability of SSL to surmount different degrees of broadening.

The line-narrowing by a factor of ca. 5 leads to a gain in signal-to-noise ratio (SNR) by a factor of ca. 5. Note the slight displacement of the line-narrowed peak (Fig. 3, dark blue line), with respect to the conventional 1D spectrum. This arises from problems in accurately determining the centre of gravity of the inhomogeneously broadened light blue line. To verify that the quantitative aspect of proton NMR is conserved when applying monochromatic SSL, we compared the integrals of the conventional 1D and line-narrowed spectra and found them to be equal.

**Figure 3:**
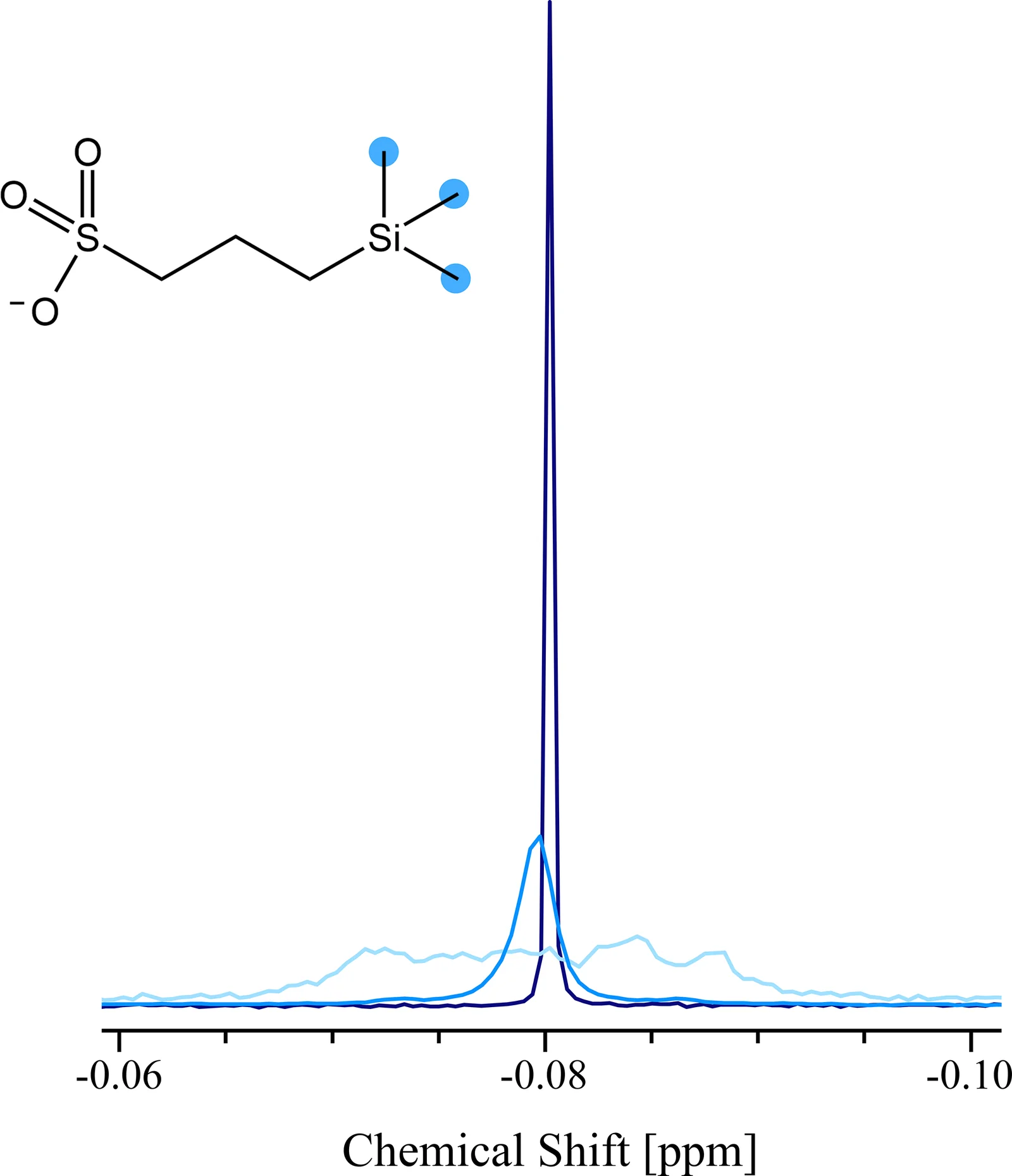
Line-narrowing by monochromatic SSL applied to the three equivalent CH_3_ groups (blue circles) of 200 µM DSS in D_2_O. Blue line: Conventional 1D spectrum with a line-width Δν=1 Hz. Light blue line (scaled ×2 ): Broadened to a line-width of about 10 Hz by deliberately mis-setting a shim current. Dark blue line: Line narrowed to Δν=0.23 Hz by monochromatic SSL applied to the broadened light blue resonance, as described in the text. The SNR is boosted by a factor ca. 5 when SSL is applied. All spectra were obtained with 64 k points and 2 scans.

## 4 Monochromatic SSL for Line-Narrowing and Decoupling of Multiplets

### 4.1 Applications to Proton NMR

#### 4.1.1 Combination with one-dimensional spectroscopy

In ^1^H NMR spectra, methylene protons (–CH_2_–) often appear as complex second-order multiplets due to couplings between magnetically inequivalent neighbouring CH_2_ protons. Here, we measured a conventional well-shimmed spectrum excited by a π/2 pulse (Fig. 4, blue line), which features a complex multiplet structure covering about 10 Hz and a central line with Δν=2 Hz. The inhomogeneously broadened peak (Fig. 4, light blue line) spans an overall width of about 50 Hz. The same monochromatic SSL experiment applied to the methyl groups of DSS, described in the previous section, was applied to the central inhomogeneously broadened CH_2_ group multiplet (Fig. 4, light blue line) of DSS giving rise to a narrow singlet (Fig. 4, dark blue line). A narrowing of the line-width to Δν=0.27 Hz, and a collapse of the multiplet are observed, boosting the SNR of the CH_2_ peak in the SSL experiment by a factor of 22.

**Figure 4:**
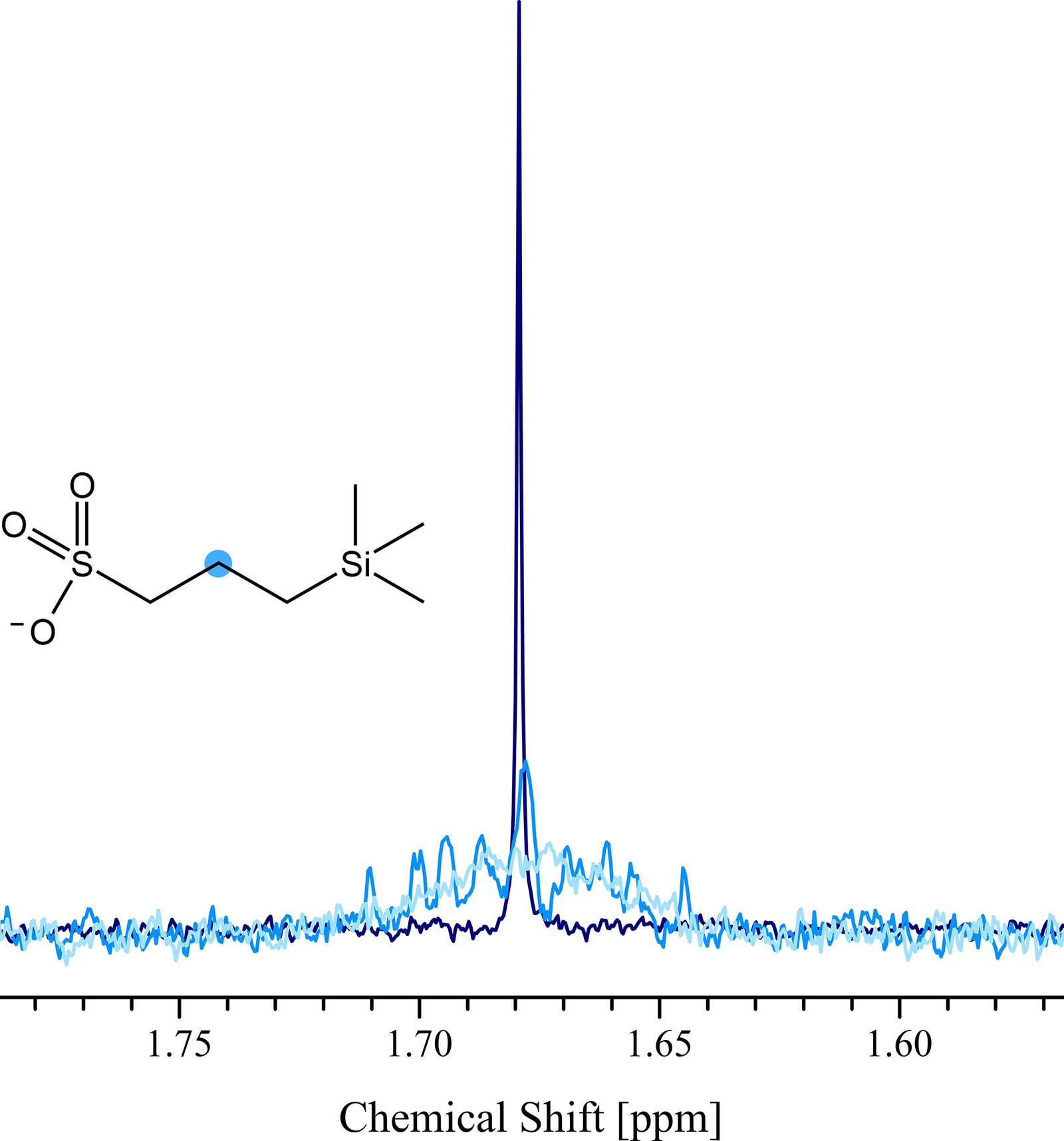
Line-narrowing and decoupling by monochromatic SSL applied to the central CH_2_ multiplet (blue circle) of 200 µM DSS in D_2_O. Blue line (scaled ×2 ): Conventional 1D multiplet with an overall width of ca. 40 Hz and Δν=2 Hz for the central line. Light blue (scaled ×2 ): Broadened by deliberately miss-setting a shim current to an overall width of ca. 50 Hz. Dark blue line: The broadened spectrum was line narrowed and decoupled to a line-width Δν=0.27 Hz by monochromatic SSL, resulting from the Fourier transformation of an FID obtained with short pulses with $\tau_{p}=2.85$ µs , applied within delays of Δt=152 µs , with an average RF amplitude of 37.5 Hz. The SNR is boosted by a factor of ca. 22 for this multiplet. All spectra were obtained with 64 k points and 2 scans.

#### 4.1.2 Combination with Long-Lived States (LLS)

In recent years, we have developed methods to excite long-lived population imbalances (Bocan et al., 2012) and reconvert them into observable magnetisation in (CH_2_)_*n*_ chains (Sonnefeld et al., 2022a) by Spin-Lock Induced Crossing (SLIC) (Sonnefeld et al., 2022b). The decays of the signals allow one to determine the lifetimes $T_{\mathrm{LLS}}$ of the superpositions of two-, four- or even six-spin order LLS terms. Applying monochromatic SSL ( $\tau_{p}=2.85$ µs , applied within Δt=152 µs , and 37 Hz average RF amplitude) to a multiplet that results from the reconversion of LLS in the low-frequency CH_2_ group of β -alanine (Fig. 5b) collapses the multiplet of an overall width equal to 18 Hz and narrows the central line-width of 1 Hz to a sharp singlet of Δν=0.3 Hz. The SNR of the peaks is increased by factors close to 3 (Fig. 5) after achieving monochromatic SSL, which is particularly welcome for these types of experiments. Indeed, at least 90 % of the signal is lost during the creation and reconversion of LLS by SLIC experiments, so that boosting the peak height by SSL offers a significant advantage. The LLS lifetime was extracted by mono-exponential fitting and was found to be $T_{\mathrm{LLS}}=17$ s, both for experiments with and without SSL.

**Figure 5:**
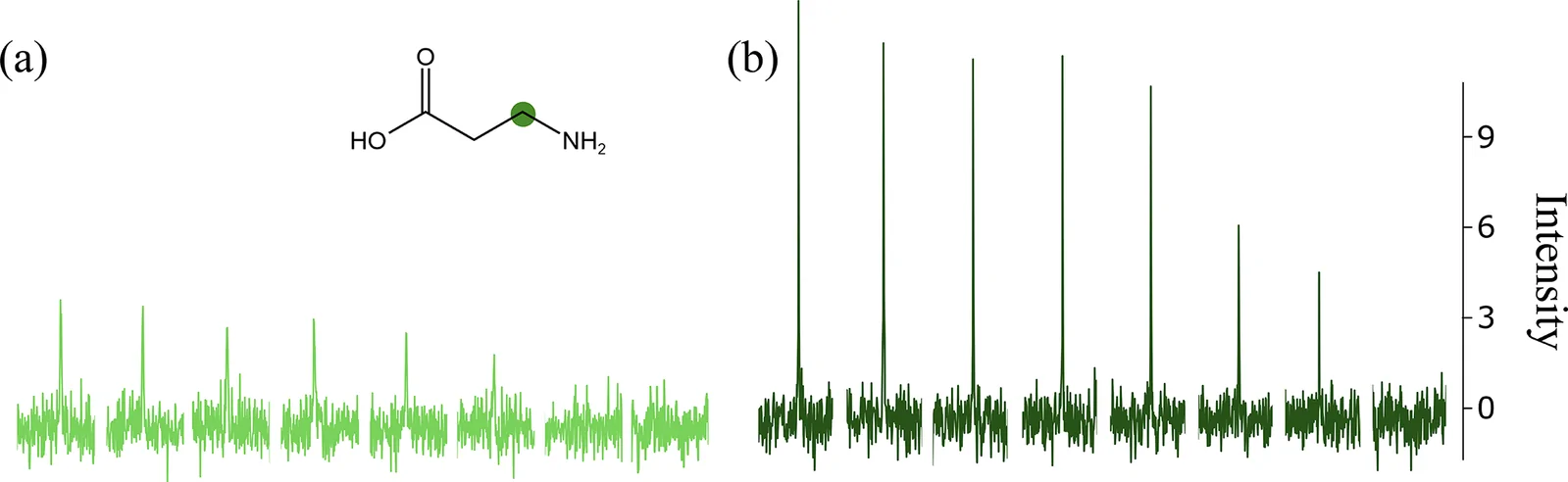
LLS signals of the low-frequency CH_2_ group (green circle) of 2 mM β -alanine in 10 mM PBS (pH 7.2) in D_2_O, obtained by the reconversion of LLS into magnetisation by SLIC. The LLS were created on both CH_2_ groups by monochromatic SLIC-in and reconverted into magnetisation by monochromatic SLIC-out. **(a)** Without SSL one observes Δν=1 Hz. **(b)** With monochromatic SSL, as described in the text, applied to the low-frequency CH_2_ group during acquisition, the line-width is narrowed to Δν=0.3 Hz. The interval between the SLIC-in and SLIC-out pulses was incremented in 8 steps of increasing size going from 0.1 to 30 s. The SNR is increased by a factor of ca. 3 when SSL is applied. Both experiments were obtained with 64 k points and 2 scans.

### 4.2 Applications to Fluorine-19 NMR

#### 4.2.1 Combination with one-dimensional spectroscopy

Fluorine-19 spectra tend to show complicated multiplets because of the ubiquity of long-range couplings. Figure 6 shows an application of a *monochromatic* SSL sequence (at 11.7 T or 470.53 MHz for ^19^F and 298 K) to perfluorobutanesulfonic acid (PFBS), a typical example of per- and poly-fluoroalkyl substances (PFAS). SSL experiments were measured on two samples of PFBS with concentrations (a) 20 mM and (b) 200 µM shown in Fig. 6. The monochromatic SSL sequence was applied, for both samples, to the central CF_2_ group of PFBS, with short pulses of $\tau_{p}=3.8$ µs (nutation frequency =2.1 kHz for ^19^F) applied within delays Δt=21.2 µs (spectral width 47.17 kHz) for an average RF amplitude of 376 Hz. These parameters were optimised to effectively collapse the entire multiplet which covers a region of ca. 76 Hz. This experiment results in a line-narrowing effect decreasing the FWHH from about 22 to 0.45 Hz for Fig. 6a, while the SNR is increased by a factor of ca. 26. For the 200 µM PFBS sample (Fig. 6b), the FWHH decreases from 22 to 0.65 Hz, while the SNR is increased by a factor of ca. 11.

**Figure 6:**
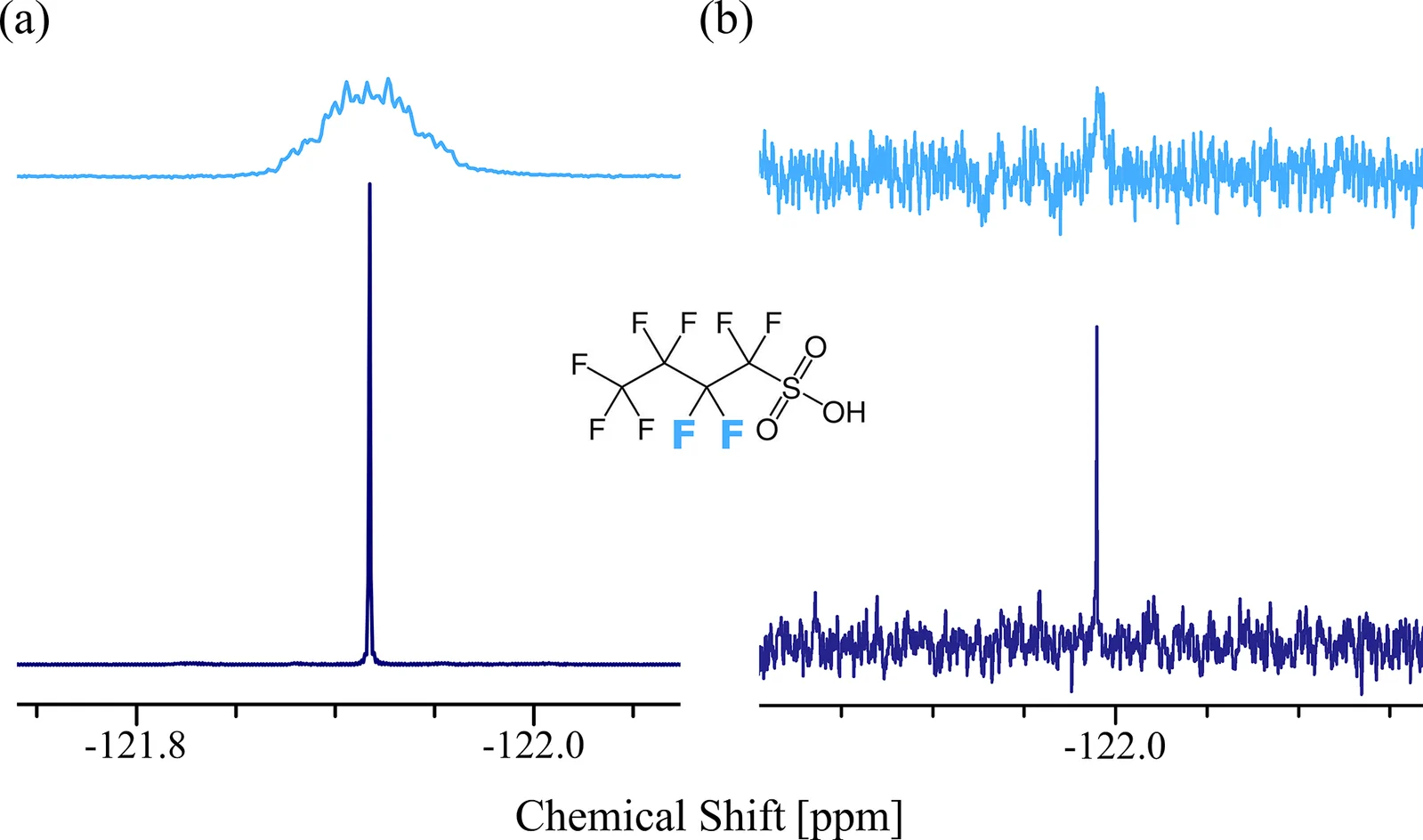
Fluorine-19 spectra of the central CF_2_ multiplet (fluorine atoms highlighted in blue) of **(a)** 20 mM and **(b)** 200 µM perfluorobutanesulfonic acid (PFBS) in D_2_O. Light blue line: Conventional 1D spectrum for **(a)**, scaled ×4 , having an overall width of about 76 Hz and a FWHH of 22 Hz and for **(b)** an overall width of about 76 Hz and FWHH of 22 Hz. Dark blue line: Line narrowed and decoupled to a line-width Δν=0.45 Hz **(a)** and for Δν=0.65 Hz **(b)** by monochromatic SSL as described in the text. The SNR is boosted by a factor ca. 26 for **(a)** and ca. 22 for **(b**). All spectra were obtained with 64 k points and 2 scans.

The noise level increases because $t_{\mathrm{RX}}$ is shorter than the dwell time. In molecules that are not perfluorinated but contain residual protons our method allows one to dispense with proton decoupling since both homo- and heteronuclear couplings are effectively suppressed.

The transverse relaxation time in the rotating frame of these ^19^F nuclei was measured by the INFERNO method (Morris and Freeman, 1978) to be $T_{1\rho }=1.39$ s, so that the narrowest theoretically possible line-width achievable by SSL for these nuclei is $\Delta \nu_{\min}=0.23$ Hz. The experimental value measured is therefore ca. 2 times larger than this limit for Fig. 6a and ca. 3 times larger for Fig. 6b.

#### 4.2.2 Combination with Long-Lived States (LLS)

In recent work, we investigated the creation and reconversion of LLS in (CF_2_)_*n*_ chains using SLIC (Wiame et al., 2025). The excitation yield of LLS in these fluorinated aliphatic chains by SLIC is only around 1 %, making SSL even more appealing than for applications to protons. Figure 7 shows the signal decay of LLS without and with monochromatic SSL ( $\tau_{p}=3.8$ µs , applied in delays Δt=42 .0 µs for an average RF amplitude of 190 Hz) irradiating the central CF_2_ peak of PFBS resulting from the reconversion of LLS. Here, the LLS signal is line narrowed and decoupled, from a line-width of 20 Hz and an overall width of ca. 57 Hz to only Δν=0.5 Hz, while increasing the SNR by a factor of ca. 20. In both experiments, it was found that $T_{\mathrm{LLS}}=3.5$ s.

**Figure 7:**
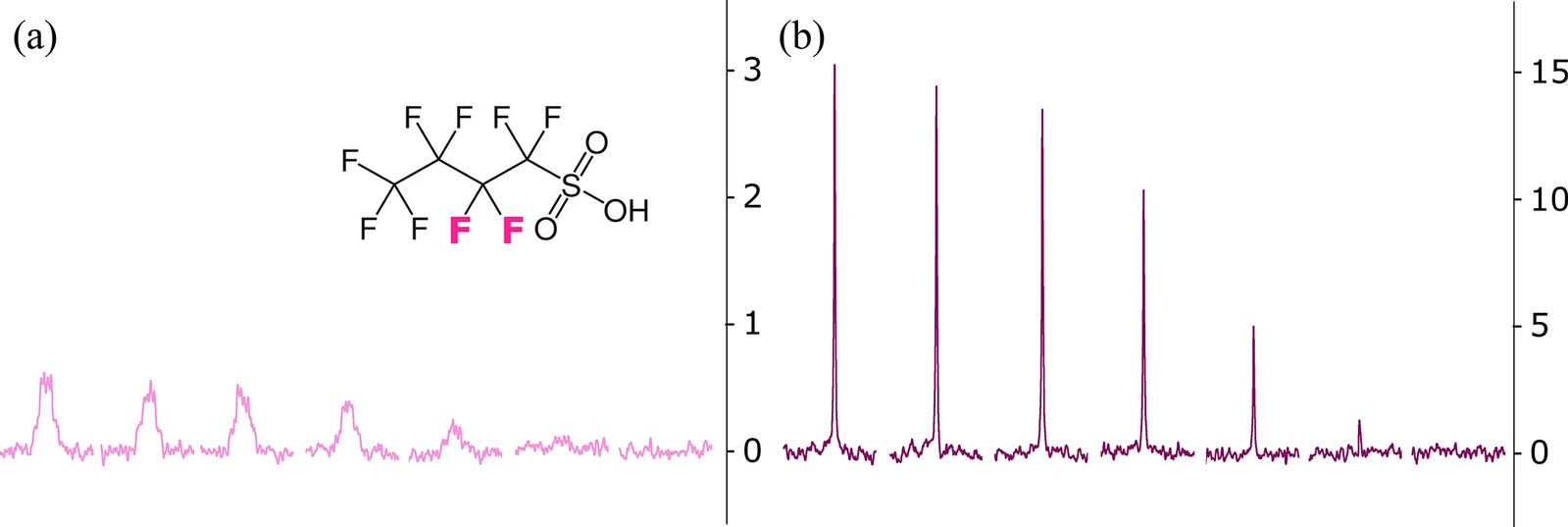
LLS signals of the central CF_2_ group (highlighted in pink) of 20 mM perfluorobutanesulfonic acid (PFBS) in D_2_O, obtained by the reconversion of LLS into magnetisation by SLIC. The LLS was created and reconverted by monochromatic SLIC-in and monochromatic SLIC-out. **(a)** Without SSL with an overall span of about 57 Hz and a FWHH of 20 Hz. **(b)** With monochromatic SSL applied to the central CF_2_ group during acquisition as described in the text, narrowing the line-width to Δν=0.5 Hz. The interval between the SLIC-in and SLIC-out pulses was incremented in 8 steps of increasing size from 0.1 to 60 s. The SNR of all signals were increased by a factor of ca. 20. Both experiments were obtained with 64 k points and 2 scans. To increase the number of data points across each line, a Lorentzian line broadening of 0.3 Hz was applied so that each peak is represented by 9 points.

## 5 Polychromatic SSL for Line-Narrowing and Decoupling

### 5.1 Applications to Proton NMR: Combination with one-dimensional spectroscopy

In addition, a *dichromatic* SSL sequence ( M=2 in Fig. 1b) was applied to both CH_2_ groups in the antibiotic metronidazole (MDZ), achieving narrowing and decoupling of both multiplets from a line-width of Δν=3 Hz for the central line and an overall width of ca. 15 Hz of the multiplet to a singlet with a line-width of only 0.23 Hz (Fig. 8). The SNR of the two peaks is increased by factors close to 38 and 31 respectively. The parameters of the dichromatic SSL are $\tau_{p}=1.9$ µs (for a nutation frequency of 1.9 kHz), applied within delays Δt=152 µs , with an average RF amplitude of 25 Hz. The carrier frequency was set half-way between the two multiplets (wavy arrow in Fig. 8) so that the effective irradiation frequencies appear at $\Delta f_{1}=-150.8$ Hz and $\Delta f_{2}=+150.8$ Hz with respect to the carrier.

**Figure 8:**
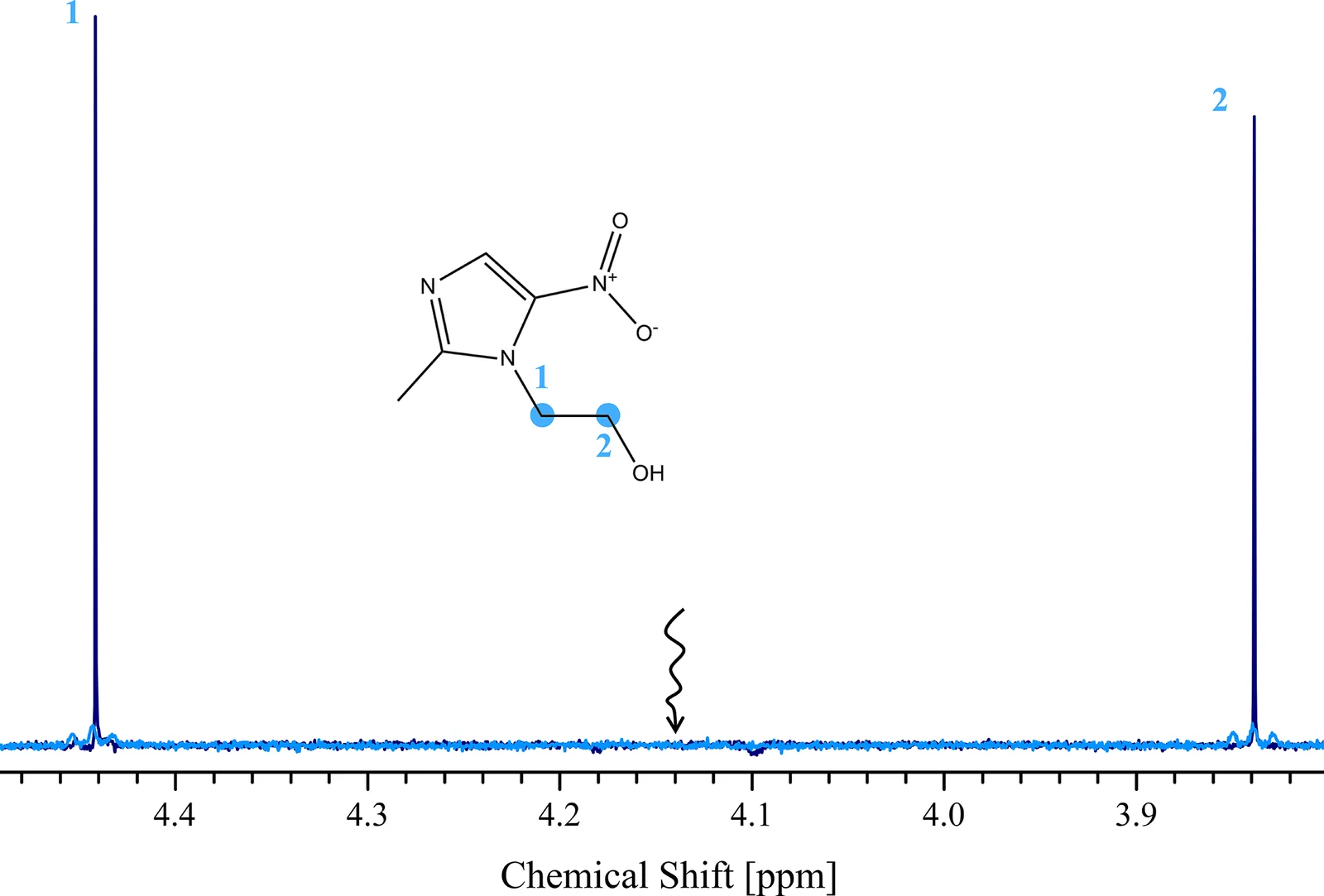
Line-narrowing and decoupling by dichromatic SSL applied simultaneously to the high- and low-frequency CH_2_ peaks (highlighted by blue circles) of 200 µM metronidazole (MDZ) in D_2_O at 11.7 T (500 MHz for ^1^H) and 298 K. Blue line: Conventional 1D spectrum with a line-width Δν=3 Hz. Dark blue line: Lines narrowed and decoupled to a line-width Δν=0.23 Hz by dichromatic SSL as described in the text. The wavy arrow indicates the RF carrier frequency. The SNR of the two peaks are increased by factors close to 38 and 31 respectively. Both spectra were recorded with 64 k points and 2 scans.

When dichromatic spin-locking is applied to the chemical shifts A and X of a coupled two-spin AX system, the effects of all couplings $J_{\mathrm{AM}}$ , $J_{\mathrm{XM}}$ to non-irradiated nuclei are suppressed, but the coupling $J_{\mathrm{AX}}$ between the two irradiated spins may not be perfectly cancelled. This effect is reminiscent of “recoupling” in solid-state experiments (Tošner et al., 2021). Furthermore, one may observe undesirable coherence transfer phenomena because of homonuclear cross-polarization if the Hartmann-Hahn condition is fulfilled. Such effects can be suppressed by using unequal average RF amplitudes of the selective fields applied to the chemical shifts A and X.

We applied an *octa-chromatic* SSL ( M=8 in Fig. 1b) sequence to a mixture of various small molecules, i.e., metronidazole, vitamin B1, acetylcholine, β -alanine, taurine and γ -aminobutyric acid (GABA), as shown in Fig. 9. In this experiment, we could effectively narrow and decouple overlapping peaks, even if their central lines were only 6.5 Hz apart. The line-widths of all peaks irradiated by octa-chromatic SSL were decreased by a factor of ca. 2, while the SNR are increased by factors ranging from 5 to 27. The same $\tau_{p}$ , average RF amplitude, and Δt used for the dichromatic SSL experiment described previously were used for octa-chromatic SSL. The carrier frequency was set to be on-resonance with the peak highlighted in yellow, corresponding to a CH_2_ group of acetylcholine (black wavy arrow in Fig. 9b) and the effective irradiation frequencies were Δ $f_{1}$ = 0 Hz, $\Delta f_{2}=+156.27$ Hz, $\Delta f_{3}=+590.30$ Hz, $\Delta f_{4}=+721.05$ Hz, $\Delta f_{5}=+363.34$ Hz, $\Delta f_{6}=-917.61$ Hz, $\Delta f_{7}=-90.34$ Hz, $\Delta f_{8}=-387.04$ Hz with respect to the carrier.

**Figure 9:**
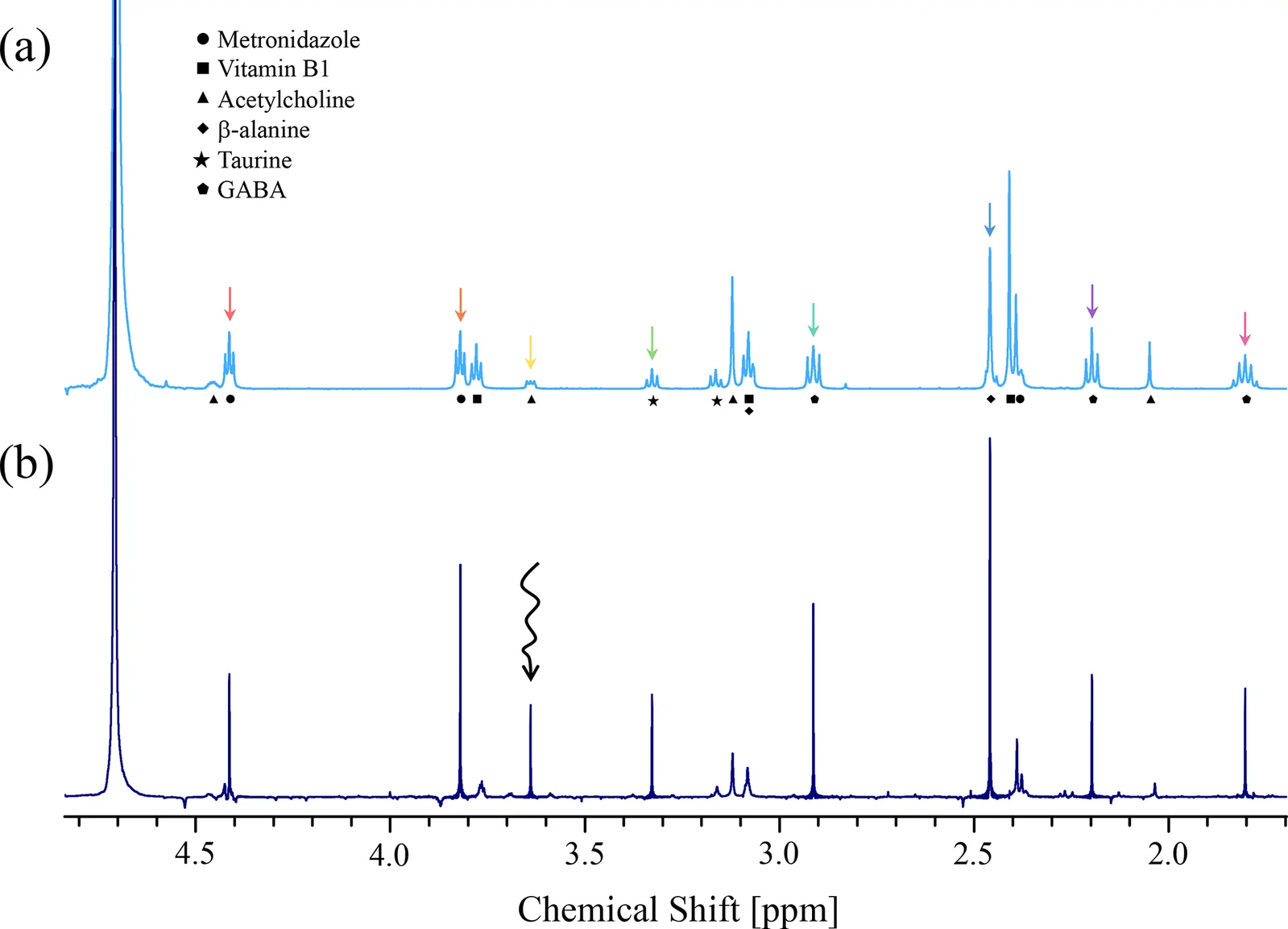
Line-narrowing and decoupling by octa-chromatic SSL applied to a mixture of six small molecules: metronidazole, vitamin B1, acetylcholine, β -alanine, taurine and gamma-aminobutyric acid (GABA), all at 10 mM concentrations in 100 mM PBS (pH 7.2) in D_2_O. **(a)** Conventional 1D spectrum, scaled ×2 . **(b)** Decoupled multiplets and narrowed lines obtained by octa-chromatic SSL as described in the text. The 8 coloured arrows in **(a)** correspond to the irradiation frequencies of the pulses chosen to spin-lock 8 selected proton signals. Symbols under the peaks in **(a)** identify to which molecules the peaks correspond. The wavy arrow indicates the RF carrier frequency. The SNR is boosted by factors between 5 and 27. To increase the number of data points across the peaks, a Lorentzian line broadening of 0.3 Hz was applied so that each peak is represented by 5 points. Both spectra were recorded with 16 k points and 2 scans.

Note that one can easily identify the Bloch-Siegert (BS) shifts (Bloch and Siegert, 1940) in the spectra of Fig. 9; for instance, the set of peaks at ca. 2.46 ppm is right-shifted by 8 Hz, 25 Hz away from the irradiation frequency (blue arrow at 2.41 ppm), which is a clear signature of a BS shift. Another set of BS-shifted peaks at 3.76 ppm are right-shifted by 7 Hz with respect to the orange arrow. The peak at 2.05 ppm is also right-shifted by 7 Hz with respect to the purple arrow.

The multiplet at 3.08 ppm is slightly left-shifted by ca. 1 Hz because it is exposed to two RF fields represented by two green arrows that are almost symmetrically disposed with respect to the multiplet.

Another observation from Fig. 9 is that the peaks that are BS shifted have strongly attenuated intensities. This can be explained by noting that the initial magnetization vector in the transverse plane can be decomposed into two components, one that is parallel to the B_1_ field and therefore spin-locked, while the component orthogonal to the effective field precesses in a plane perpendicular to this inhomogeneous field. Therefore, these signals are both frequency-shifted and attenuated.

Quantitative comparisons between conventional and SSL spectra also require consideration of the steady state established during repeated scans. In a conventional 1D experiment, the interval available for longitudinal recovery is the recovery delay plus acquisition period, because recovery towards equilibrium also occurs during acquisition. In an SSL experiment, the RF pulses applied during the acquisition can perturb longitudinal magnetisation as it recovers, so that the effective recovery interval for the irradiated spins is limited to the recovery delay. Consequently, when it is not sufficiently long compared with $T_{1}$ , the conventional and SSL experiments may establish different steady states, leading to an attenuation of the SSL integrals.

Another important consideration is that during SSL acquisition, the receiver is blanked during the RF pulses and associated dead-time intervals, and each complex FID point is derived from the signal acquired during the remaining observation window. This interrupted acquisition reduces the effective observation time and may decrease the signal-to-noise ratio or introduce instrumental scaling and modulation artefacts. To first order, such a receiver-window effect should act as a common factor for all resonances and therefore cannot by itself explain selective attenuation of individual signals. A systematic separation of these effects using calibrated windowed reference experiments is left for future work.

### 5.2 Proton NMR: Combination with Inversion Recovery (IR)

We were also able to combine *dichromatic* SSL with the Inversion Recovery ( $T_{1}$ ) experiment, as shown in Fig. 10b for the amino acid β -alanine. The line-widths of the peaks are ca. 1 Hz and the overall width of the multiplet is about 23 Hz, it is reduced to Δν=0.25 Hz by dichromatic SSL. The SNR is increased by 11 and 8 for the orange and blue spectra respectively. The $T_{1}$ was extracted by mono-exponential fitting and found to be ca. 2.7 s, both without and with SSL. Adding an SSL block after an IR sequence therefore provides a robust method for measuring $T_{1}$ values while boosting the peak heights by a sizable factor.

**Figure 10:**
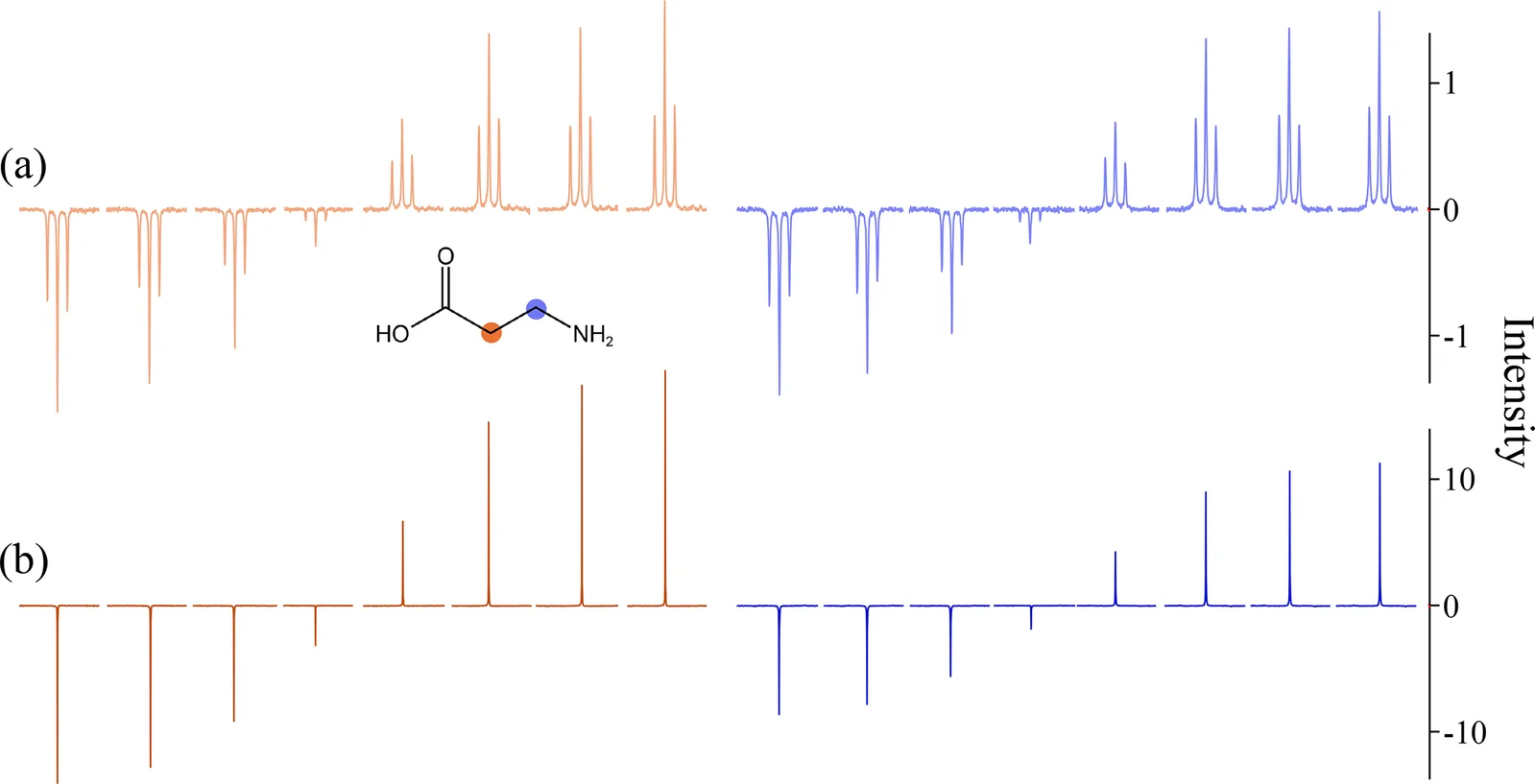
Inversion-Recovery ( $T_{1}$ ) experiments showing the high-frequency CH_2_ (orange) and low-frequency CH_2_ (blue) groups of 2 mM β -alanine in 10 mM PBS (pH 7.2) in D_2_O. **(a)** Without SSL with a line-width Δν=0.8 Hz. **(b)** Lines narrowed and decoupled to line-widths close to Δν=0.24 Hz by dichromatic SSL as described in the text. The SNR is increased by 11 and 8 for the orange and blue spectra, respectively. The interval between the inversion pulse and the excitation pulse was incremented in 8 non-linear steps going from 0.1 to 30 s. Both experiments were obtained with 64 k points and 2 scans.

Likewise, $T_{2}$ CPMG spin-echo experiments can benefit from SSL. Such experiments (not shown in this work) were run and yielded accurate relaxation times $T_{2}$ .

**Figure 11:**
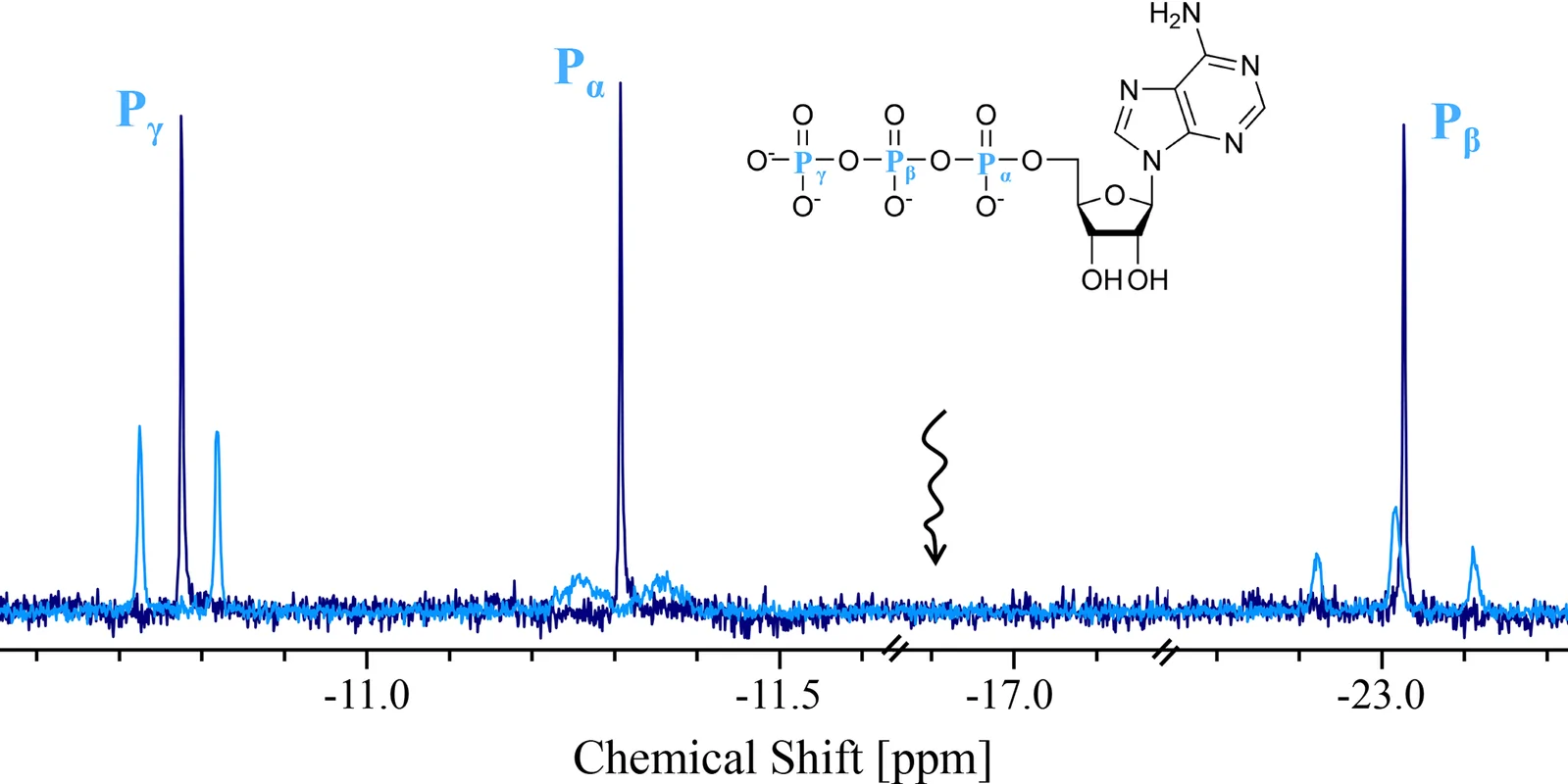
Phosphorus-31 spectra of 20 mM ATP (phosphorus atoms highlighted in blue) in D_2_O at 11.7 T (202.41 MHz for ^31^P) and 298 K. Blue line: Conventional ^31^P spectrum. Black line: Line-narrowed and decoupled spectrum obtained by trichromatic SSL, as described in the text, with line-widths Δν=0.7 Hz at all three sites. The wavy arrow indicates the RF carrier frequency. Both spectra were obtained with 64 k points and 2 scans.

## 6 Applications to Phosphorus-31 NMR

Although phosphorus-31 resonances at high fields are broadened due to rapid relaxation driven by the chemical shift anisotropy, which cannot be eliminated by SSL, various other line-broadening mechanisms are amenable to line-narrowing. To assess this, we looked at the three ^31^P nuclei in Adenosine Triphosphate (ATP). In addition to the decoupling of homonuclear J (^31^P, ^31^P) interactions, a significant line-narrowing effect was observed (Fig. 11). In the conventional spectrum, the individual lines of the left-hand doublet have widths of about Δν=1.3 Hz, and lines of the right-hand triplet have Δν=2.3 Hz. The two doublets span widths of about 20 Hz while the triplet spans a width of ca. 40 Hz. By applying *trichromatic* SSL to all three chemical shifts, the multiplets are decoupled and their lines are narrowed to 0.7 Hz. One can notice a significant enhancement of the SNR for P_*α*_ compared to the other signals which arises from the decoupling of hetero-nuclear coupling with neighbouring protons which broaden this doublet. Note that the noise level is increased in the SSL experiments of Fig. 11 by a factor of ca. 2. The optimised parameters for this experiment were $\tau_{p}=3.8$ µs , Δt=152 µs and $\langle \omega_{1}/(2\pi )\rangle =35$ Hz. The carrier frequency was set half-way between the outer multiplets at - 16.9 ppm (black wavy arrow in Fig. 11). The irradiation frequencies were $\Delta f_{1}=-1239.9$ Hz, $\Delta f_{2}=-1132.3$ Hz and $\Delta f_{3}=+1239.9$ Hz. The fact that the three peaks have nearly the same heights suggests that SSL yields quantitative intensities, i.e., that the peak heights are proportional to the numbers of spins and hence to the concentrations.

The transverse relaxation time in the rotating frame of the central ^31^P nucleus was measured by the INFERNO method (Morris and Freeman, 1978) to be $T_{1\rho }=0.357$ s, so that the narrowest theoretically possible line-width achievable by SSL for these nuclei is $\Delta \nu_{\min}=1/\pi T_{1\rho }=0.89$ Hz. The experimental line-widths were even narrower since a linewidth Δν=0.7 Hz is measured at all three sites.

## 7 Conclusions

By applying mono- or polychromatic SSL to selected singlets or multiplets in high-resolution NMR spectra of ^1^H, ^19^F, ^31^P and other nuclei, one can collapse both homo- and heteronuclear scalar couplings, eliminate inhomogeneous broadening, and thus obtain very narrow signals. The decay of the spin-locked transverse magnetisation is in principle limited by $\mathrm{exp}\{-t/T_{1\rho }\}$ rather than by $\mathrm{exp}\{-t/T_{2}^{*}\}$ , so that the line-widths upon Fourier transformation can approach the theoretical limit $\Delta \nu_{\min}=1/\pi T_{1\rho }$ rather than the inhomogeneous width $1/\pi T_{2}^{*}$ .

The reduction of line-widths and decoupling of multiplets leads to an increase in peak heights, ranging from factors 4 to 40 in the examples discussed in this work. This should make it possible to reduce the detection level, i.e., the minimum concentration that is required to detect the NMR signals, provided the identity of the molecules under investigation and their chemical shifts are known. This may be of interest for metabolomic studies.

The SSL method should also be useful for special probes where several samples can be studied in parallel, where the static homogeneity and hence the line-widths may suffer from imperfect shimming (Van Dyck et al., 2026).

Applications of SSL are not limited to small molecules. The line-widths in proton spectra of macromolecules at high fields are usually dominated by homogeneous $T_{2}$ relaxation, and $T_{1\rho }$ is usually close to $T_{2}$ rather than $T_{1}$ as in small molecules, so that line-narrowing by SSL is not likely to be very effective. However, decoupling of homo- and heteronuclear scalar interactions (Cavanagh et al., 2007) such as J (^1^H, ^1^H), J (^13^C, ^1^H) and J (^15^N, ^1^H) may be useful in macromolecules. Furthermore, the lines can be broadened by chemical exchange, in particular by exchange between major and minor conformers, and the effects of SSL should be investigated empirically.

All magnetic resonance imaging (MRI) methods achieve spatial resolution by using pulsed field gradients, including “frequency encoding” during signal observation. Spin-locking should obviously not be applied during signal observation (Mosher and Smith, 1990). However, in a sub-class of MRI experiments that have come to be known as magnetic resonance spectroscopy (MRS), where the signals are observed in the absence of gradients to determine the chemical shifts, spin-locking can be applied during signal observation. This can be used inter alia to determine the concentrations of various constituents or metabolites in the brains of rodents and humans, as well as phosphorylated molecules like ATP (Chung et al., 2024), phosphocreatine, etc., in various organs. At high fields, the line-widths in phosphorus-31 spectra are usually dominated by homogeneous $T_{2}$ relaxation, particularly if the chemical shift anisotropy (CSA) is the dominant line-broadening mechanism. A priori, such line-widths are not amenable to narrowing by SSL. However, we have shown that significant line-narrowing can be achieved in addition to decoupling of J (^31^P, ^31^P) interactions. Furthermore, the line-widths of ^1^H, ^31^P or ^13^C resonances in vivo are broadened by discontinuities in tissues, variations in susceptibility, and motions due to arterial or respiratory effects. Such static or dynamic line-broadening effects should be largely mitigated by mono- or polychromatic SSL during signal acquisition.

In Magic Angle Spinning (MAS) solid-state NMR, the line-widths in proton spectra are usually dominated by residual dipole-dipole interactions (Chávez et al., 2021) that cannot be suppressed by SSL. However, the line-widths in MAS spectra of ^13^C and other dilute nuclei are often broadened by disorder in amorphous or polycrystalline materials. Their line-widths could therefore be narrowed by SSL, in as far as the modulations due to sample spinning do not interfere with the short pulses used in SSL. Synchronisation of these pulses with the rotor period can alleviate such problems (Caravatti et al., 1983).

In solids spinning at the magic angle, half-integer spins like ^7^Li and ^23^Na feature central transitions between the m=±1/2 levels that are broadened by inhomogeneous second-order quadrupole interactions. Such line-shapes could be collapsed to their average positions by applying SSL near the frequency of their first moment. A superposition of M overlapping sites (as occurs in battery materials) could be separated by a two-dimensional method where M -chromatic SSL is applied in the detection period but not in the evolution interval.

The SSL method might be applicable to other two-level systems such as occur in optical spectroscopy, although broadening by Doppler effects might not be amenable to line-narrowing.

A wide range of methods collectively known as “pure shift spectroscopy” have been derived from two-dimensional J-spectroscopy (Aguilar et al., 2010; Castañar and Parella, 2015). These ingenious methods allow one to collapse multiplets due to scalar couplings. While the final result bears similarities to mono- or polychromatic SSL, in particular since decoupling is accompanied by line-narrowing (in principle limited only by the homogeneous line-width $1/\pi T_{2}$ for pure shift spectroscopy), the mechanisms are quite distinct, and mono- or poly-chromatic spin-locking is more efficient in terms of signal-to-noise per unit time. Similar considerations can be applied to the SHARPER method (Jones et al., 2017; Davy et al., 2022; Dickson et al., 2022). By contrast, the DREAMTIME method (Jenne et al., 2022) uses doubly selective irradiation tailored for known compounds.

## Data Availability

The pulse sequences used for the SSL experiments and some of the spectral data in this manuscript are available at 10.5281/zenodo.22305292 (Wiame et al., 2026).
